# Revealing Interfacial Reactions on Pt Electrodes in Ionic Liquids by In Situ Fourier-Transform Infrared Spectroscopy

**DOI:** 10.1021/acs.analchem.3c02903

**Published:** 2023-10-30

**Authors:** Yingzhen Chen, Christian Rodenbücher, Klaus Wippermann, Carsten Korte

**Affiliations:** † Institute of Energy and Climate Research—Electrochemical Process Engineering (IEK-14), Forschungszentrum Jülich GmbH, 52425 Jülich, Germany; ‡ RWTH Aachen University, 52062 Aachen, Germany

## Abstract

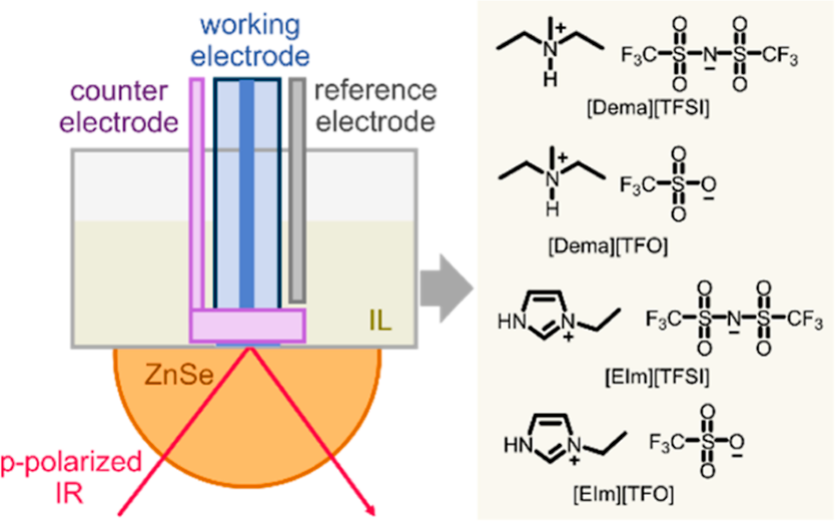

In situ monitoring of the electrolyte/electrode interfacial processes, such as the oxygen reduction reaction (ORR), is crucial for the design of electrolytes for fuel cells. In this study, we investigate the electrochemical behavior of platinum electrodes in protic ionic liquids (PILs) by means of in situ Fourier-transform infrared spectroscopy coupled with cyclic voltammetry. The result provides direct evidence of the change of water at the Pt electrode surface due to Pt oxide formation and reduction. A decrease in the interfacial water was observed in the spectra upon the formation of the Pt oxide. Conversely, the local water concentration at the electrode surface increases if the Pt oxide is reduced and the ORR takes place. At the same time, more cations replace anions on the electrode. The ORR kinetics in the [TFSI]-based PILs is slower than in the [TfO]-based ones, which could result from a blockage of catalytic sites by the adsorbed [TFSI] anions. It suggests that reducing the anion adsorption on the platinum surface could provide an opportunity to enhance the ORR activity.

## Introduction

Polymer electrolyte fuel cells (PEMFCs) have emerged as promising candidates to replace combustion engines in automotive applications. However, PEMFCs using sulfonated fluoropolymers, e.g., NAFION or AQUIVION, whose proton conduction relies on the presence of water, have limited operating temperatures of below 80 °C (ambient pressure). The operation of a PEMFC at elevated temperatures above 100 °C would enable a significantly simplified system setup for water and heat management. Operating temperatures of 100–120 °C require novel nonaqueous electrolytes. Protic ionic liquids (PILs) have been suggested as proton carriers for PEMFCs because of their small vapor pressure and high chemical and thermal stability.^1−3^

Ionic liquids (ILs) usually consist of large and bulky organic cations and anions of strong acids/superacids, resulting in a small lattice energy and, thus, a melting point below 100 °C. A PIL usually consists of a cation or anion with an acidic proton. In this study, we will focus on PILs [BH][A] with an acidic cation BH^+^, which can be prepared by a protolytic reaction between an organic base B and a super acid HA (B + HA → BH^+^ + A^–^). A large variety of combinations of cations and anions provide the opportunity to design electrolytes with the desired properties. Therefore, it is crucial to understand the structural properties of ILs, which affect the oxygen reduction reaction (ORR) activity. The acidic cation BH^+^ in these PILs can act as the proton donor and carrier. The proton availability on the cation has been considered one of the most important effects on the ORR activity. Cations with different acidities (p*K*_a_) were investigated and improvement in the onset potential of ORR has been observed in the order of increasing cation acidity.^4,5^ The open-circuit potential of H_2_/O_2_ is also reported to be related to Δp*K*_a_ between the cations and anions. Either too high or too low Δp*K*_a_ will decrease the ORR activity.

Understanding the ORR mechanism at the molecular level is very important for the further enhancement of the ORR activity. In situ monitoring techniques such as Raman/infrared spectroscopy (IR) and atomic force microscopy (AFM) have been used to extensively study the electrode/electrolyte interface during electrochemical processes,^6−12^ which has contributed to great progress in the understanding of ORR kinetics in aqueous electrolytes. Neat PIL electrolytes consist of only charged species, which leads to a more complex electrical double-layer structure close to the electrode surface compared with classical aqueous electrolytes. Alternating densely packed anion and cation layers have been found, e.g., by AFM, close to a charged electrode, which has also been supported by molecular dynamics simulations. In situ Raman and IR spectroscopies reveal the ion adsorption behavior on Ag and Au electrode surfaces as a function of the applied potential. In spite of these advancements, the interfacial structure of PILs and their interaction, especially with the catalytic electrode surface (e.g., Pt), have not been analyzed in detail so far.

**Figure 1:**
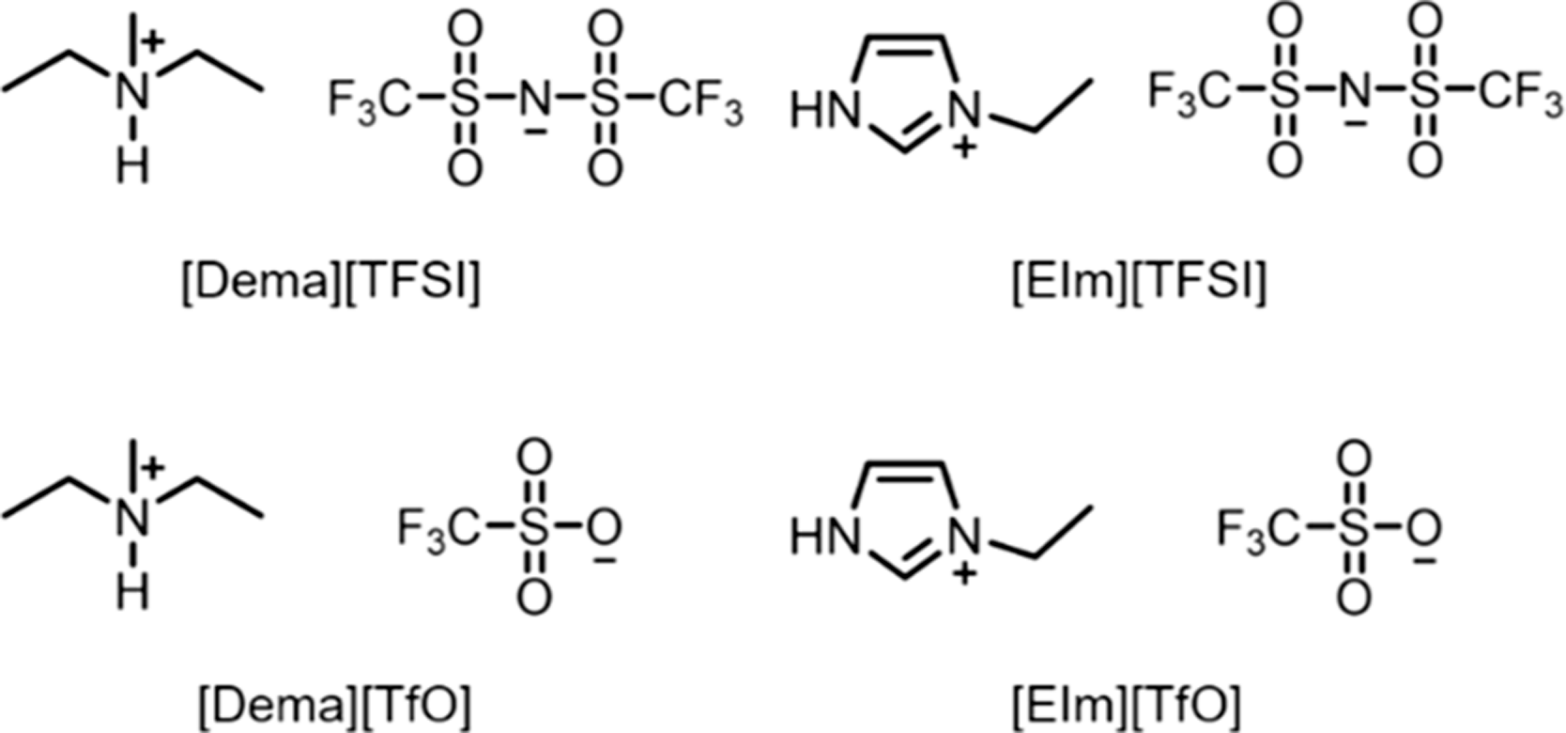
Chemical structures of PILs: [Dema][TFSI], [Dema][TfO], [EIm][TFSI], and [EIm][TfO].

Herein, we investigated the electrolyte/electrode interfacial processes of polycrystalline platinum in PILs by means of in situ Fourier transform infrared (FTIR) spectroscopy, coupled to cyclic voltammetry (CV). Four PILs, namely, diethylmethylammonium triflate [Dema][TfO], diethylmethylammonium bis(trifluoromethanesulfonyl)imide [Dema][TFSI], 1-ethylimidazole triflate [EIm][TfO], and 1-ethylimidazole bis(trifluoromethanesulfonyl)imide [EIm][TFSI] were investigated (Figure 1). Our results reveal the interfacial reactions at the Pt electrode in the PILs and the corresponding potential-dependent structural changes of the electric double layer.

## Experimental Section

### Ionic Liquids

[EIm][TfO], [EIm][TFSI], and [Dema][TfO] (>98%) were purchased from IoLiTec (Germany) and used as received. For the synthesis of [Dema][TFSI], *N*,*N*-diethylmethylamine was added dropwise to bis(trifluoromethanesulfonyl)imide (70% aqueous solution, 1.05 equiv) under ice cooling for about 0.5 h. A second, hydrophobic phase emerged. After stirring at room temperature for 2 h, the aqueous phase was removed and the residual hydrophobic phase was dried in a vacuum at 80 °C for 3 days. The resulting IL was confirmed by NMR spectroscopy. The bis(trifluoromethylsulfonyl)-imide aqueous solution (>70%) was also purchased from IoLiTec (Germany). *N*,*N*-Diethylmethylamine (>97%) was purchased from Sigma-Aldrich (USA). The water content was determined via Karl Fischer titration, as depicted in Table S1.

### Electrochemical IR Spectroscopy

**Figure 2:**
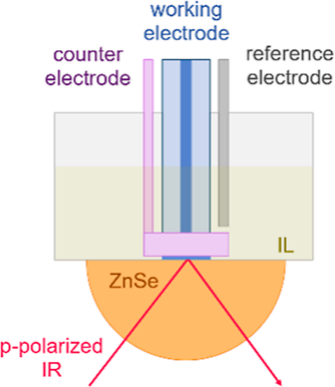
Schematic illustration of the electrochemical IR setup.

In situ infrared spectroscopy was performed by using an FTIR spectrometer equipped with an HgCdTe detector (Bruker Vertex 70v, Germany). An in-home-built electrochemical cell with a ZnSe hemisphere as the optical window was used for the measurements (Figure 2). The electrochemical measurements were performed using a BioLogic SP-300 potentiostat (France). A hydrogen-saturated palladium wire having a fixed potential of 50 mV vs RHE at 25 °C in aqueous solutions served as a reference electrode (RE), which will be referred to as the “Pd–H electrode” in the following. The Pd–H electrode has been used as a RE^13−16^ in order to avoid possible contamination by foreign ions originating from REs of the second kind, such as Ag/AgCl. In case of concentrated solutions like phosphoric acid or ILs with residual amounts of water and temperatures between RT and 130 °C, the potential difference between the Pd–H electrode and the RHE is small, ranging between ≈0 and ≈20 mV.^16^ Due to this reason and the fact that measurements of the potential difference between the Pd–H electrode and a reference hydrogen electrode are rather tedious because the equilibration time required is at least 1 h per experiment and has to be performed for each IL, temperature, and water content, we have chosen to indicate the electrode potential vs. Pd–H electrode. A platinum mesh with an area of 3.2 cm^2^ was used as the counter electrode (CE). A (polycrystalline) platinum wire with a diameter of 1 cm was employed as the working electrode (WE). The electrode was polished with an alumina suspension, followed by sonication in an ultrasonic bath at room temperature and drying under N_2_. Estimation of the active surface of the electrode was performed by cycling between potentials of 0 and 1.5 V vs Pt–H in a 0.5 M H_2_SO_4_ solution as reported in the literature.^17^ The charge related to hydrogen adsorption was determined by integrating the observed current. The real surface area of the Pt electrode was estimated to be 2.03 ± 0.19 cm^2^, which corresponds to a roughness factor of 2.6. The electrolytes were saturated with oxygen for 1 h before the measurements, which were then carried out under an oxygen atmosphere at a flow rate of 30 mL min^–1^. The FTIR spectra were measured using p-polarized light in an external reflection at an angle of incidence of 60°. All spectra were obtained with a spectral resolution of 2 cm^–1^, and every spectrum was acquired by averaging a total of 36 scans. Thus, each IR spectrum was collected within 35 s. The measurements were carried out in continuous scan mode during the CV experiment. The scan rate of the CV was adjusted to 2 mV/s, thus each IR spectrum was measured within an electrode potential range of 0.07 V. Thus, increasing the number of scans (of the IR signal) to obtain the IR spectrum will increase the accuracy and reproducibility of the spectroscopic data. On the other hand, it would increase the electrode potential range to which a single IR spectrum can be assigned to. Thus, an average of 36 scans of the IR signal was selected.

## Results and Discussion

### CV of ILs with a Pt Electrode

**Figure 3:**
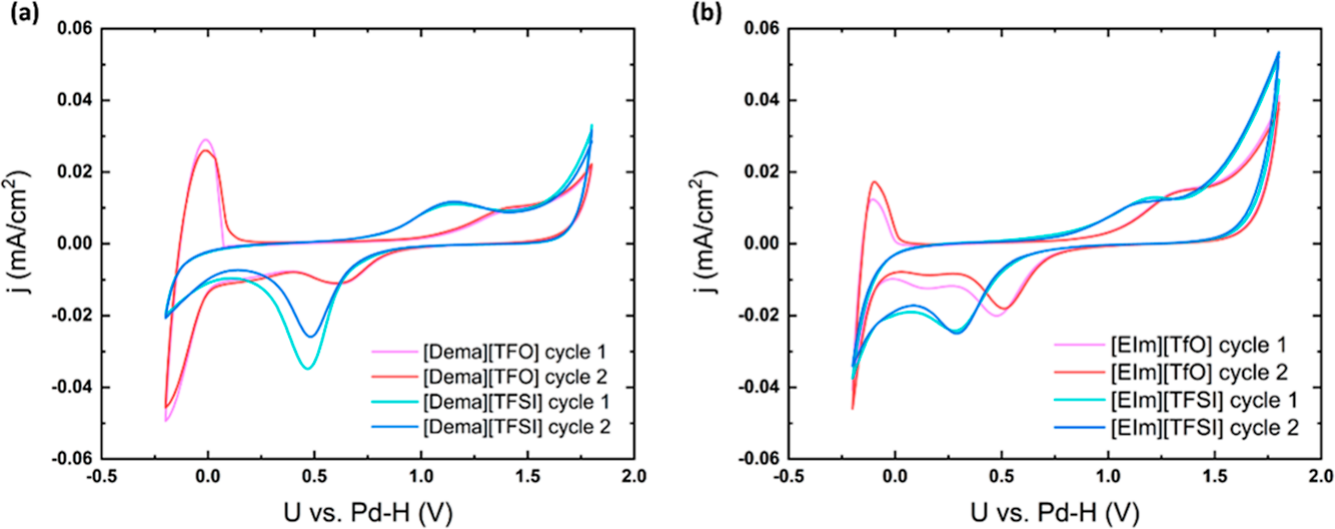
CV of ILs (a) [Dema]-based ILs; (b) [EIm]-based ILs in O_2_ with a Pt electrode at a scan rate of 2 mV/s.

Figure 3 displays the characteristic CV curves of a Pt electrode in the neat ILs, which were obtained in situ by cycling the potential between −0.2 and 1.8 V in an O_2_ atmosphere. Similar shapes of CV are observed in two different cycles of CV scans. The CVs of PILs based on the same anion exhibit a similar shape with respect to the characteristics of features due to Pt-oxidation/reduction and to O_2_ and H_2_ oxidation/reduction. The same redox feature can be found for [Dema][TfO] and [EIm][TfO], whereas the shape of the curves of [Dema][TFSI] and [EIm][TFSI] is very similar.

The potential region between −0.2 and 0.1 V shows interesting characteristic features. In [TfO]-based ILs, the reduction current increased during the negative sweep below 0 V and an oxidation wave was found during the subsequent positive sweep. Thus, the signal observed for the negative sweep below 0 V may be regarded as the consequence of an underpotential deposition of hydrogen (H_UPD_) on the platinum surface and the signal at the positive sweep as the subsequent reoxidation and desorption from the surface. This coincides well with the CV curves of Pt electrodes in aqueous H_2_SO_4_ solutions. However, anodic peaks below 0 V have not been observed in the [TFSI]-based PILs, i.e., [Dema][TFSI] and [EIm][TFSI]. This indicates that the hydrogen evolution/H_UPD_, as well as the reoxidation and desorption on Pt, respectively, can only be observed in [TfO]-based ones but not in [TFSI]-based ILs when the potential cycling is limited to −0.2 V. According to Ejigu and Walsh,^18^ this results from a blockage of the H adsorption sites by [TFSI] anions on the Pt surface.

The oxidation wave above 1 V and the reduction peak below 0.9 V can be attributed to platinum oxidation and oxide reduction. In the case of [Dema][TfO], platinum oxidation starts at around 1 V and the current density reaches a plateau at about 1.4 V. These signals can be attributed to the oxidation of the platinum surface, forming a thin layer. This phenomenon is known for diluted aqueous acidic electrolytes and can be described according to the following reaction sequence
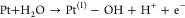
1a
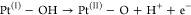
1b
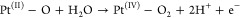
1c

The formation of a PtOH layer can be observed in aqueous acidic electrolytes at 0.9 V. The PtOH layer is oxidized to PtO and subsequently to PtO_2_ with an increasing positive potential. A thickness on the order of 10 Å can be found in aqueous electrolytes.^19^ According to the given reaction sequence, water is required for the formation of platinum oxide. A low concentration of water could even be found in samples of (quasi) neat ILs. In particular, PILs tend to absorb water from the air due to their high polarity. In addition, H_2_O can be produced electrochemically during ORR in the cathodic region of the CV scan. Thus, an increase in the water content is found following the measurements (Table S1). A small amount of water at the electrode is sufficient to allow the formation of a thin platinum oxide surface layer. During the reverse scan toward negative potentials, the platinum oxide layer is reduced, leading to a reduction current starting at 0.89 V and reaching its maximum at 0.62 V. In the case of aqueous acidic electrolytes, a current peak is found at about 0.8 V.^19^

In comparison to the current signals of platinum oxidation and the reduction in [Dema][TfO], those in [EIm][TfO] shift slightly toward lower values. For a comparison of the PILs, the onset potential refers to the potential at which the current density reaches 0.002 mA/cm^–1^. In the case of [EIm][TfO], the onset potentials of the platinum oxidation and reduction are found at 0.90 and 0.79 V, which is about 0.1 V lower than that in [Dema][TfO]. The maximum of the oxidation peak is located at 1.25 V for [EIm][TfO] and 1.37 V for [Dema][TfO]. Two reduction peaks are observed in [TfO]-based PILs: the first, larger peak occurs at 0.48 V for [EIm][TfO] and at 0.62 V for [Dema][TfO]; the second, smaller peak appears at 0.14 V for [EIm][TfO] and 0.24 V for [Dema][TfO], respectively. These two reduction steps are also found in [Dema][TfO], including 90% water as well as other [TfO]-based PILs.^4,20^ According to Wippermann et al.,^4^ the first reduction peak is due to the common Pt-oxidation/oxide reduction mechanism via hydronium ions as proton donors (see eqs 1a–1c), whereas the second may be assigned to a mechanism whereby the cation acts as a proton donor as well. The redox peaks of the [TFSI]-based PILs generally shift to lower potentials. The oxidation peaks of [EIm][TFSI] and [Dema][TFSI] are observed at 1.13 and 1.16 V; the corresponding reduction peaks are located at 0.32 and 0.50 V, respectively. In contrast to [TfO]-based PILs, the second reduction peak is not visible in the [TFSI]-based PILs, indicating that the cation may not be involved in the reduction process.

The oxygen evolution reaction (OER) due to water electrolysis is observed at potentials of >1.5 V. This proves the presence of interfacial water in both hydrophilic and hydrophobic PILs. The resulting OER reduces the electrochemical window of the PILs. The [EIm]-based PILs exhibit higher OER current densities compared to the [Dema]-based ones.

### In Situ IR Spectra of the Pt/IL Interface

**Figure 4:**
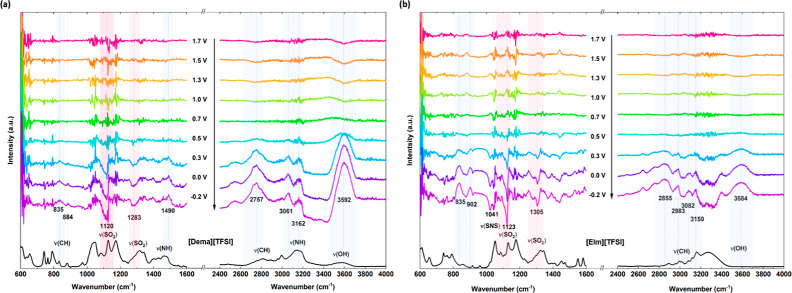
Potential-dependent IR spectra of (a) [Dema][TFSI] and (b) [EIm][TFSI] recorded during a cathodic scan at a scan rate of 2 mV/s. The reference spectrum was taken at 1.8 V before the scan. The spectra of bulk PILs are shown in the black curve at the bottom.

**Figure 5:**
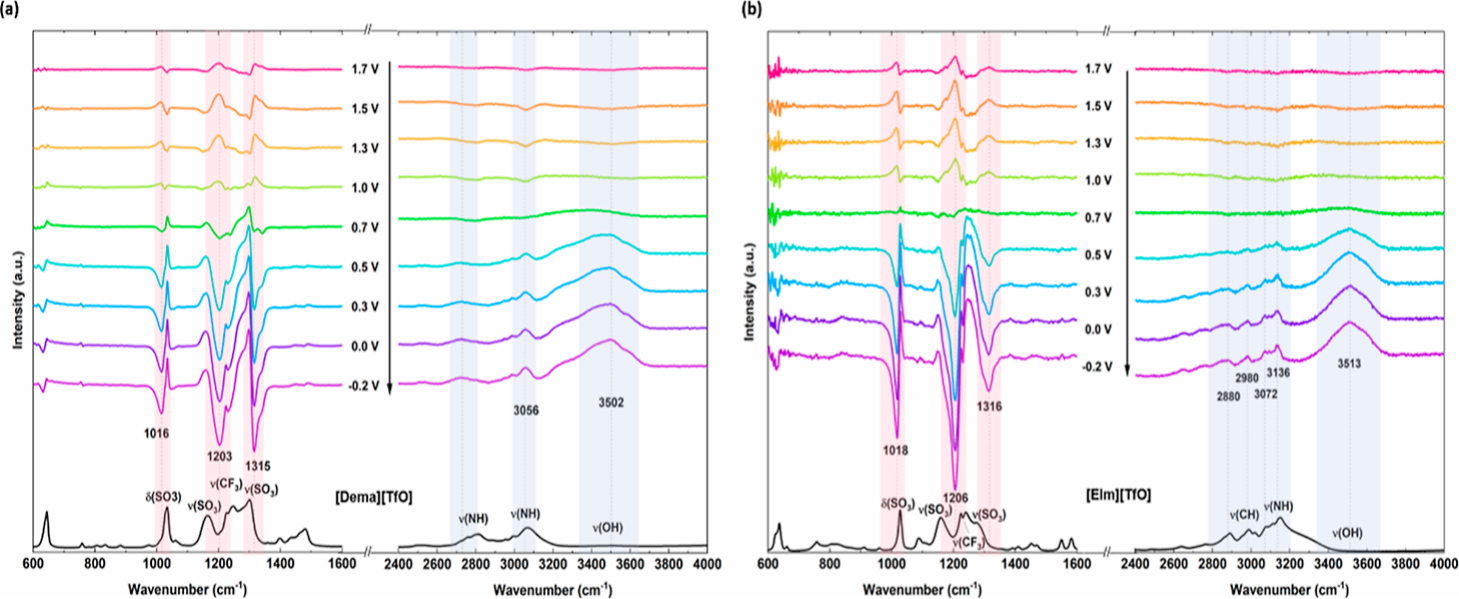
Potential-dependent IR spectra of (a) [Dema][TfO] and (b) [EIm][TfO] recorded during a cathodic scan at a scan rate of 2 mV/s. The reference spectrum was taken at 1.8 V before starting the scan. The spectra of bulk PILs are shown in the black curve at the bottom.

To further understand the electrochemical interaction of various PILs with a Pt electrode, we collected in situ IR spectra during CV cycling. A series of spectra measured in the oxygen-saturated PILs are depicted in Figures 4 and 5. The spectra are plotted in absorbance units according to the equation: *A* = log(*I*_0_/*I*), where *I* is the intensity in the sample spectrum and *I*_0_ is a reference spectrum.^21^ In order to observe the dynamic changes of the PILs at the electrode surface as a function of potential, a reference spectrum *I*_0_ was acquired at a cell potential of 1.8 V. In this case, a positive, upward-pointing peak corresponds to an increasing intensity due to a surface accumulation or local formation of the species assigned to this vibration mode compared to the reference. A negative, downward-pointing peak indicates decreasing intensity due to a surface depletion or local consumption of the species.

For comparison, the IR spectra of the bulk PILs [Dema][TFSI], [Dema][TfO], [EIm][TFSI], and [EIm][TfO] are illustrated as black curves below the potential-dependent IR spectra in Figures 4 and 5, as well as in Figure S1 in the Supporting Information. For the spectra of the bulk PIL, reference *I*_0_ is the intensity measured without an PIL. The most prominent bands of the [TfO]^−^ and [TFSI]^−^ anions appear in the spectral range of 1000–1400 cm^–1^. As the anion vibration modes dominate in intensity, the ILs with the same anion show similar characteristic peaks. The bands related to the cations are less intense in the low wavenumber region, whereas the cation modes are prominent in the range of 2500–3400 cm^–1^.

A broad peak detected above 3400 cm^–1^ can be related to the ν(OH) stretching vibration of water molecules dissolved in the ILs. In the case of [Dema][TFSI], the ν(OH) stretching mode of water adsorbed at the Pt electrode can be observed as a band at 3592 cm^–1^, as shown in Figure 4a. The band appears upon decreasing the cell potential below 0.5 V. It increases rapidly up to 0.3 V. At a more negative potential, the increase slows down. In the case of [EIm][TFSI], shown in Figure 4b, the broad peak of the ν(OH) stretching vibration is found at a value of 3584 cm^–1^. In the triflate-based PILs [Dema][TfO] and [EIm][TfO] (see Figure 5), however, the ν(OH) peak is observed at significantly lower wavenumbers of 3502 and 3513 cm^–1^, respectively, compared to that of the bistriflimide-based PILs. Water tends to form more hydrogen bonds with the anions than the cations,^22^ which causes a weakening of the covalent water O–H bond and a decrease in the frequency. The lower wavenumber of the ν(OH) in the triflate-based PILs indicates that water has a stronger interaction with [TfO]^−^ than [TFSI]^−^. In addition, the difference in vibration frequencies could also result from different orientations of water molecules on the electrode surface. According to previous studies, the peaks at about 3600 cm^–1^ in [TFSI]-based PILs can be assigned to the dangling O–H bonds of the interfacial water, whereas those at 3500 cm^–1^ in [TfO]-based PILs suggest trihedrally coordinated water.^10^

The vibration modes of the anion are located in the range of 1000–1400 cm^–1^. As depicted in Figure 4a, an intense negative-going band can be observed at a cell potential below 0.5 V in [Dema][TFSI] at 1120 cm^–1^ and another relatively weak one is located at 1283 cm^–1^. In the spectra of [EIm][TFSI] shown in Figure 4b, analogously sharp negative-going peaks are visible at 1123 and 1305 cm^–1^ at cell potentials below 0.3 V. These bands can be assigned to the overlap of the symmetrical stretching mode ν_s_(SO_2_) and the asymmetrical stretching mode ν_as_(SO_2_) of the [TFSI]^−^ anion. In the low-wavenumber region of [TfO]-based PILs, three characteristic negative-going peaks can be noticed in Figure 5. The peaks are situated at 1016, 1203, and 1315 cm^–1^ in the case of [Dema][TfO] and at 1018, 1206, and 1316 cm^–1^ in that of [EIm][TfO]. The peak at 1018 cm^–1^ can be assigned to the symmetric stretching ν_s_(SO_3_). The s-sharp feature of the signal could result from the peak shifting to lower frequencies. As indicated in the spectra of the bulk PILs, the peaks in the 1100–1350 cm^–1^ range are due to the overlapping stretching modes of the triflate group, i.e., the asymmetric stretching ν_as_(SO_3_), symmetric stretching ν_s_(CF_3_), and asymmetric stretching ν_as_(CF_3_). The downward-pointing bands indicate a decrease in the intensity of either the sulfonyl or triflate group from the [TfO]^−^ anion as the potential decreases from 0.5 to −0.2 V.

In the range of 2600–3400 cm^–1^, only vibration modes of the cation can be observed. In the other regions, the cation bands are comparably weak and partially overlap with the bands assigned to the anion. Four upward peaks are visible at 2855, 2983, 3082, and 3150 cm^–1^ in the spectra of [EIm][TFSI] in Figure 4b. They are relocated to 2880, 2980, 3072, and 3136 cm^–1^ for [EIm][TfO]. The peaks could be assigned to CH and NH stretching of the [EIm]^+^ cation. In the case of [Dema][TFSI], the stretching vibration modes of the [Dema]^+^ cation are present at 2757, 3061, and 3162 cm^–1^, whereas only small peaks are found at 3056 and 2982 cm^–1^ in the case of [Dema][TfO]. In order to identify the assignments of the vibration modes of the [Dema]^+^ cation, in situ IR investigations were performed in deuterated [Dema][TfO], i.e., with a (C_2_H_5_)_2_CH_3_N-D^+^ cation under the same condition. In addition to the peak of the ν(OD) stretching mode at 2597 cm^–1^, we found two additional peaks at 2094 and 2264 cm^–1^, which can be identified as being caused by ν(ND) stretching. They become apparent when the potential is below 0.5 V (Figure S2). This evidence supports the contention that these vibration modes with increasing intensities for positive cell potentials can be assigned to the cation.

**Figure 6:**
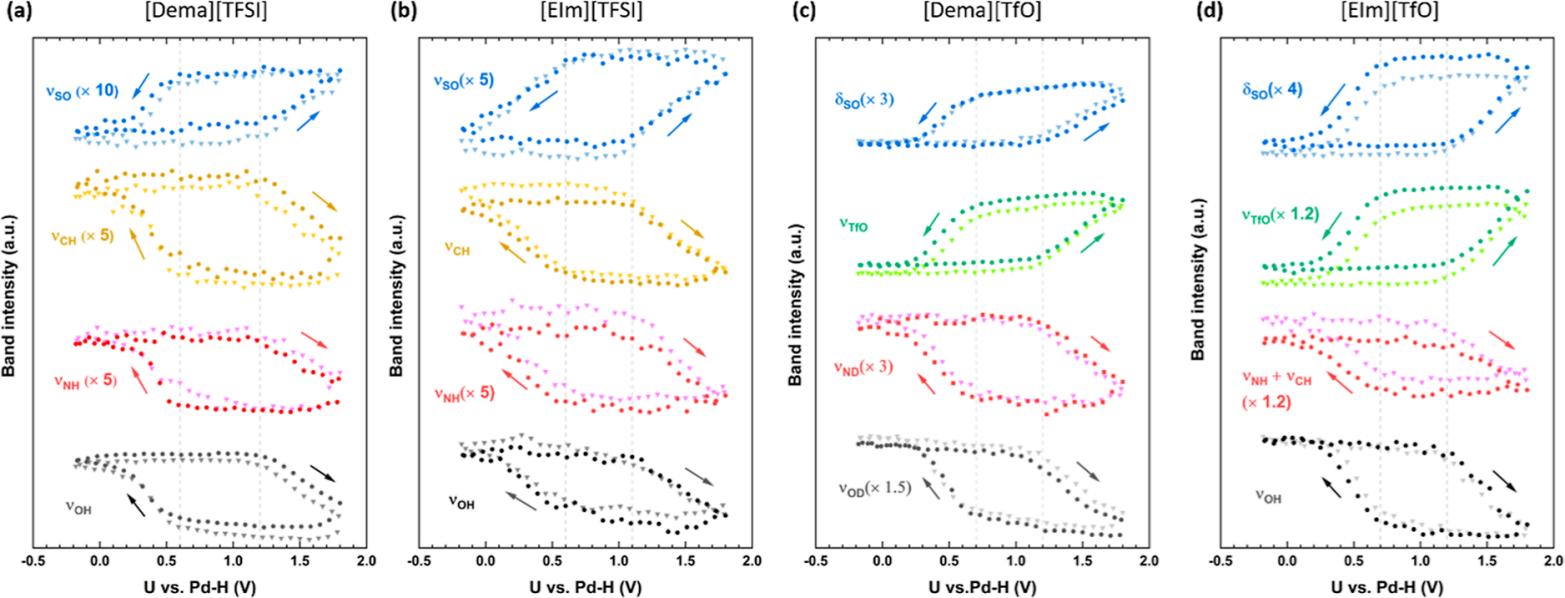
Integral intensities of various IR bands of (a) [Dema][TFSI]; (b) [EIm][TFSI]; (c) [Dema][TfO]; and (d) [EIm][TfO] as a function of potential during the two potential scan cycles between −0.2 and 1.8 V. The first cycle is indicated by triangles; the second cycle is noted by circles.

In order to gain a direct view of the potential-dependent behavior at the electrode surface, the intensities of the vibrational peaks were integrated and plotted as a function of the potential. Figure 6 illustrates the results for two cycles of potential scans between −0.2 and 1.8 V. The characteristic bands at 3592, 3162, 2757, and 1120 cm^–1^ found in the spectra of [Dema][TFSI] and those at 3584, 3150, 2855, and 1123 cm^–1^ in the spectra of [EIm][TFSI] are plotted to trace the change of ν(OH), ν(CH), ν(NH), and ν(SO). In the spectra of the [TfO]-based PILs, the peaks at 1016 cm^–1^ in [Dema][TfO] and 1018 cm^–1^ in [EIm][TfO] are labeled δ(SO). The broad peaks at ca. 1200 cm^–1^, which correspond to the overlapping bands of ν(SO) and ν(CF), are colored green and marked as ν(TfO). The total intensities of the overlapping peaks at around 3000 cm^–1^ in the spectra of [EIm][TfO] are integrated to show the ν(NH) and ν(CH) modes from [EIm]^+^. As the signals from the vibration modes of the cation in [Dema][TfO] are weak, the intensities of ν(ND) and ν(OD) from the (C_2_H_5_)_2_CH_3_N-D^+^ cation in the spectra of deuterated [Dema][TfO] are used to trace the potential-dependent change in the interfacial cation concentration.

On the positive-going sweep starting from −0.2 V, none of the band intensities change significantly until 1.2 V for [Dema][TFSI], [Dema][TfO], [EIm][TfO], and 1.1 V for [EIm][TfO], respectively. As the potential becomes more positive, the intensity of ν(OH) stretching mode drops steeply for all of the investigated PILs, together with the increase in the anodic current in the CV due to Pt surface oxidation (Pt + *x* H_2_O → Pt–O_*x*_ + 2H^+^ + e^–^). If the ν(OH) stretching mode is ascribed to adsorbed water molecules on the Pt surface, this means a decrease in the coverage of water. A straightforward explanation for this is the consumption of water by the Pt-oxidation process, even though more water tends to be adsorbed on the electrode at more positive potentials.^22,23^ The decreased intensities of the ν(OH) stretching mode indicate that the consumption of water proceeds faster than the electrosorption process.

On the reverse scan, the intensities of ν(OH) increase gradually until 0.6 V for the [TFSI]-based ILs and 0.7 V for the [TfO]-based ones. For more negative cell potentials, the intensities increase more steeply and, finally, only gradually again. The significantly increased signal of ν(OH) is attributed to more water accumulating at the electrode. This could result from water produced from the reduction of Pt–O_*x*_ + 2H^+^ + e^–^ → Pt + *x*H_2_O and O_2_ + 4H^+^ + e^–^ → 2H_2_O. The interfacial water could in turn form H_3_O^+^, which would serve as the proton donor for ORR. Furthermore, the ORR kinetics would be improved because more catalytic sites would be available on the reduced, blank Pt surface. This is supported by the observation of Pt nanoparticles and the roughness of the electrode surface by in situ AFM in an aqueous acidic solution.^6^

For all of the investigated PILs, the change in the FTIR intensities of cations and anions is always accompanied by the platinum redox reaction, as depicted in Figure 6. The opposite change in the intensities of the cations and anions revealed that the cation–anion replacement takes place on the electrode surface. When the potential increases above 1.2 V, the intensities of the cation vibration modes decrease, whereas those of the anion vibration modes increase. This indicates that anions, replacing cations, adsorb increasingly on the electrode surface. In the negative potential scan, the cation-to-anion replacement starts from 0.7 V in [TfO]-based ILs and from around 0.6 V in the [TFSI]-based ones. The delayed replacement of the anions could be attributed to different anion adsorption strengths, with [TFSI]^−^ anions interacting more strongly with the Pt surface than [TfO]^−^ anions.^18^ A stronger adsorption of [TFSI]^−^ would result in a larger kinetic barrier for platinum reduction and, by blocking active sites, would also inhibit the ORR. The adsorption strengths can be altered by variations of fluoroalkyl chain lengths {H–N[SO_2_(CF_2_)_*n*_F]_2_}, as reported in previous FTIR studies.^24^ Tunable anion adsorption on the platinum surface could provide a new opportunity to enhance the ORR activity. Several studies have been devoted to controlling ORR activity by tuning the proton activity (p*K*_a_) using a more acidic cation,^4,13,15,16,25,26^ which could lead to an increase in local proton activity near the active sites. [EIm]^+^ cations have higher acidity than the [Dema]^+^ cations; however, the adsorption of [TFSI]^−^ anions hinders the access of oxygen and protic cations to the catalytic sites of the electrode surface. This is supported by the observation of a missing second reduction process with the cations as the proton donor in the case of the [TFSI]-based PILs in the CV curves in Figure 3. Our results suggest that the anion plays a dominant role and becomes the limiting factor of ORR activity.

A significant potential difference between the cation and anion replacements was observed in this study, leading to a pronounced hysteresis in the band intensity vs. potential. This hysteresis was also observed in previous studies of surface-enhanced infrared absorption spectroscopy of aprotic ILs on a gold electrode surface.^27^ With decreasing scan rates, the hysteresis behavior becomes more pronounced, suggesting that it is caused by the overpotential for overcoming the energy barrier of restructuring.^27^ Our study reveals that the oxide layer on the electrode surface hinders electrocatalytic performance and so imposes a higher energy barrier. This contributes to the hysteresis behavior of ion replacement on PILs at the Pt surface. It has been reported by Huang et al. that the formation of surface oxide and the ordering of water dipoles could generate a negative charge on the oxide surface.^28,29^ Because a cation-to-anion replacement is observed at potentials above 1.2 V, i.e., in the potential region of oxide formation, this may suggest that the negative charge due to surface oxide dipoles cannot compensate for the strong Coulomb forces of the highly positively charged electrode. However, a continuous substitution of cations by anions can also be explained by a negative charge whose absolute value decreases with increasing potential, i.e., a surface charge vs. potential characteristic that was proposed by Huang for elevated potentials.^28^

## Conclusions

In this study, we investigated the electrochemical behavior of polycrystalline platinum electrodes in PILs by means of in situ FTIR coupled to CV. The presence of interfacial water was found for both hydrophilic and hydrophobic PILs. The change in the interfacial water and ion concentration was associated with Pt oxide formation and reduction. The consumption or generation of interfacial water during the oxidation and reduction process can be monitored by an anodic/cathodic current flow in CV and a decrease/increase in the intensity of the O–H vibration mode in FTIR spectroscopy. Simultaneously, the replacement of cations by anions and vice versa was verified by a change in the intensity of the ion-specific bands. Compared to [TFSI] anions, [TfO] anions tend to form more hydrogen bonds with water, which causes a weakening of the covalent water O–H bond. This interaction may also change the orientation of the water molecules at the electrode.

Moreover, the results reveal that the adsorption of the PIL anions onto a catalytic electrode surface clearly has an important influence on the electrochemical properties. The more positive onset potential of the ORR in the [TfO]-based PILs compared to the [TFSI]-based ones suggests a stronger interaction of the [TFSI] anion with the Pt surface, eventually blocking catalytic sites and hindering the access of oxygen, hydronium ions, and protic cations. Conversely, [TfO]-based PILs provide a lower overpotential of the ORR on platinum and so a better cathode performance. Therefore, altering the anion adsorption on the platinum surface could provide an opportunity to enhance the ORR activity.

## Supporting Information Available

IR spectra of bulk PILs, potential-dependent IR spectra of deuterated [Dema][TfO], and water content of PILs before and after measurements (PDF)
