# Supplementary material for: Revealing Interfacial Reactions on Pt Electrodes in Ionic Liquids by In Situ Fourier-Transform Infrared Spectroscopy
Source: Anal Chem. 2023 Oct 30;95(45):16618–24. doi: 10.1021/acs.analchem.3c02903 (PMC10652234; doi:10.1021/acs.analchem.3c02903)
Supplement: Supplementary file 1 — ac3c02903_si_001.pdf [file ac3c02903_si_001.pdf]

Supporting Information

Revealing interfacial reactions on Pt electrodes in ionic liquids by *in-situ* Fourier-transform infrared spectroscopy

Yingzhen Chen,[a,b] Christian Rodenbücher,[a] Klaus Wippermann,[a] and Carsten Korte*[a,b]

[a] Institute of Energy and Climate Research – Electrochemical Process Engineering (IEK-14), Forschungszentrum Jülich GmbH, 52425 Jülich, Germany

[b] RWTH Aachen University, 52062 Aachen, Germany

*Email: c.korte@fz-juelich.de

Table of content

Figure S1 IR spectra of bulk ionic liquids: (a)[Dema]-based ILs;(b)[EIm]-based ILs.··················S2

Figure S2 Potential-dependent IR spectra of deuterated [Dema][TfO] recorded during a cathodic scan at a scan rate at 2 mV/s. The spectra of bulk ionic liquids are marked by the black curve at the bottom. ··S3

Table S1 Water content of PILs before and after measurements. ···································S3

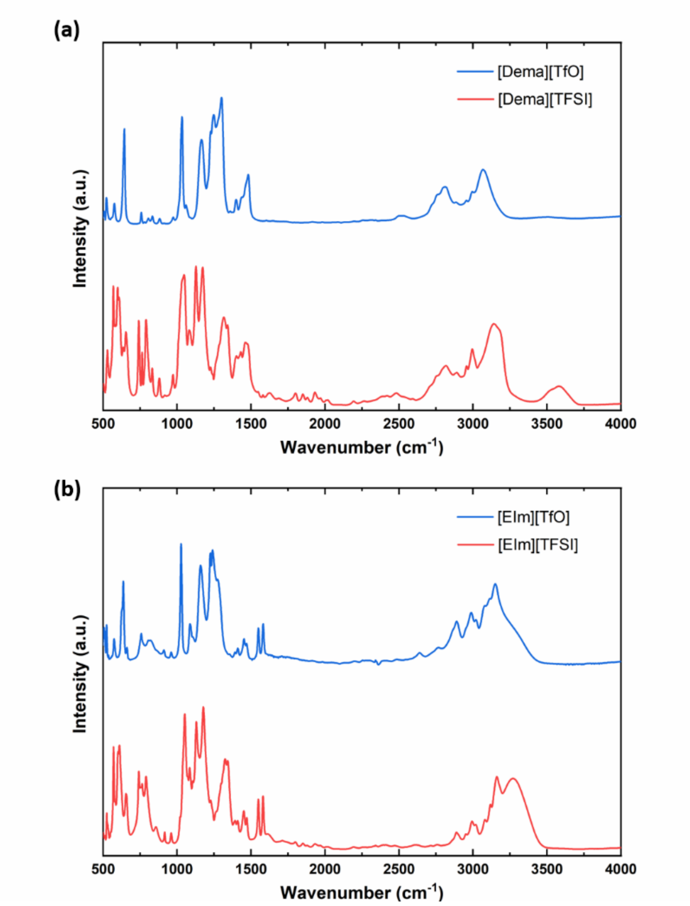

Figure S1 IR spectra of bulk ionic liquids: (a)[Dema]-based ILs;(b)[EIm]-based ILs.

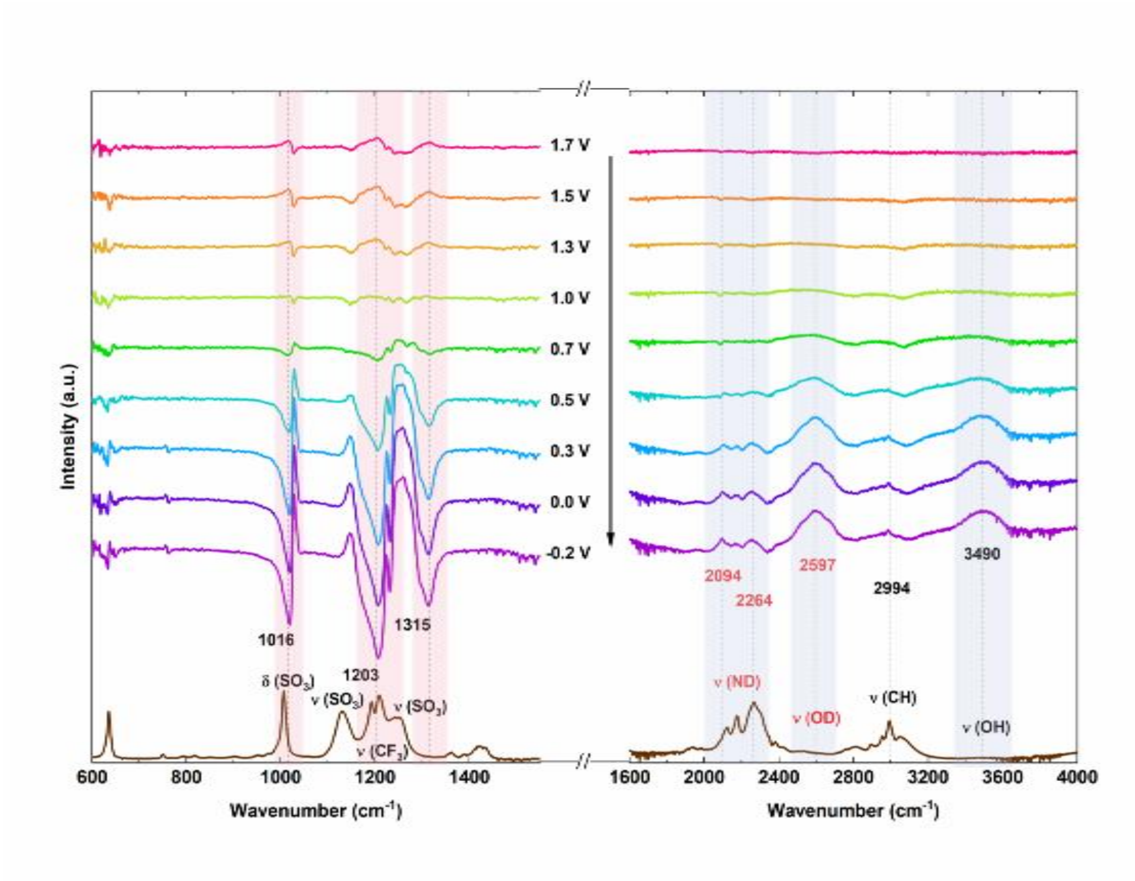

Figure S2 Potential-dependent IR spectra of deuterated [Dema][TfO] recorded during a cathodic scan at a scan rate at 2 mV/s. The spectra of bulk ionic liquids are marked by the black curve at the bottom.

Table S2 Water content of PILs before and after measurements.

Water content (wt%)	[Dema][TFSI]	[EIm][TFSI]	[Dema][TfO]	[EIm][TfO]
Before measurements	0.11 ± 0.04	0.03 ± 0.003	0.35 ± 0.01	0.08 ± 0.01
After measurements	0.82 ± 0.001	0.63 ± 0.001	0.93 ± 0.01	1.09 ± 0.01
